# Bibliometric Analysis for Carbon Neutrality with Hotspots, Frontiers, and Emerging Trends between 1991 and 2022

**DOI:** 10.3390/ijerph20020926

**Published:** 2023-01-04

**Authors:** Guofeng Wang, Rui Shi, Wei Cheng, Lihua Gao, Xiankai Huang

**Affiliations:** 1 Faculty of International Trade, Shanxi University of Finance and Economics, Taiyuan 030006, China; 2 Institute of Geographic Sciences and Natural Resources Research, Chinese Academy of Sciences, Beijing 100101, China; 3 Office of Scientific Research & Development, Beijing Technology and Business University, Beijing 100048, China; 4 School of Mathematics and Statistics, Beijing Technology and Business University, Beijing 100048, China

**Keywords:** carbon neutrality, bibliometric analysis, Citespace, Vosviewer, hotspots, frontiers

## Abstract

The proposal of carbon neutrality is a manifestation of actively responding to global warming and sustainable development, which means all greenhouse gases achieve near-zero emissions. China is also fulfilling its national mission in this regard. This paper collected 4922 documents from the “Web of Science Core Database” and used Citespace (6.1.R2 Advanced) and Vosviewer (1.6.18) software and Bibliometrix functions to carry out descriptive statistics on the number of publications, cooperation mechanisms, and keyword hotspots, finding that the literature mainly focused on China’s carbon neutrality, carbon emissions, energy efficiency, sustainable development, and other related topics in the past two years. Further, the 2060 carbon neutrality action plan for China is discussed, focusing on the implementation plan and technical route and proposing the corresponding plans. The purpose of this paper is to accelerate the pace of China’s achievement of this goal and to provide feasible solutions and pathways to its achievement through insight into global carbon neutrality hotspots and new trends.

## 1. Introduction

Climate change has posed a huge threat to human survival and development, ranging from transnational trade to cellular respiration, as carbon emissions are everywhere. The full implementation of the Paris Agreement (2015) and the Statement of the Carbon Neutrality Alliance (2017) ushered in the era of carbon neutrality and set off a climax of international “carbon” actions [1,2]. In 2020, the global surface temperature has moved upward about 1.2 °C compared with the pre-industrial period, accompanied by the frequent occurrence of extreme climatic events, and the temperature is increasing at a rate of about 0.19 °C per year [3]. Researchers from the Intergovernmental Panel on Climate Change (IPCC) believe that the global average temperature will climb until at least the middle of this century, given the current situation [4]. Projections indicate that the temperature control target of less than 2 °C set by the Paris Agreement will be reached in 2065; thus, the task of achieving carbon neutrality is urgent. According to Net Zero Tracker data, 136 countries/regions attach great importance to carbon neutrality and have proposed the goal of achieving net-zero emissions in the second half of this century [5]. These goals cover 88% of global emissions, 90% of gross domestic product, and 85% of the world population. Asia, known as the “engine of the world economy”, accounts for more than half of the world’s total carbon emissions, of which China was the country that produced the most carbon emissions in 2021. The Chinese government has proposed the 2060 carbon neutrality goal; that is, China needs to achieve its goal in only 30 years (2030–2060), equivalent to the timespan of up to 40 years (2007–2050) in the USA and 60 years (1990–2050) in the EU [6,7]. The challenge of achieving carbon neutrality is thus greater and harder. As a result, issues related to carbon neutrality have been given high priority by research institutions and scholars in China, but they mainly focus on the institutional level and fail to systematically and comprehensively review and present a full picture of carbon neutrality.

Carbon neutrality has been under consideration for more than 30 years. It refers to individuals or organizations achieving “net-zero carbon emissions” through afforestation and other means, but this concept does not take into account that “carbon dioxide emissions must be emitted” until carbon neutral technologies are widely adopted [8,9]. It is now widely recognized in academic circles that carbon neutrality is requires a balance between the rates of emission of anthropogenic carbon emissions (carbon sources) and natural (or other) carbon sequestration (carbon sinks) such that the concentration of carbon dioxide in the atmosphere will no longer increase [10]. The carbon neutrality formula is as follows:

Carbon emissions = ocean absorption + carbon sequestration in ecosystems + anthropogenic carbon sequestration + carbon sequestrations by other surface processes

Looking back, governments and international institutions around the world have made carbon neutrality a focal point and a long-term challenge for climate action. Europe is currently playing an important role in promoting carbon neutrality and formulating many technical routes and policy measures to promote the world’s carbon reduction work [11]. It has accumulated considerable mature practical experience in low-carbon technologies, carbon recovery and social transformation. Countries continue to strengthen their emission reduction determination and efforts, and they actively submitted the long-term low greenhouse gas emission development strategy (LTS) by the end of 2020 [12]. The LTS is an important roadmap for countries to jointly achieve carbon neutrality goals [13]. Research institutions and scholars in various countries have put a new premium on carbon neutrality. Wu et al. compared the carbon neutrality policies and carbon emission reduction stage achievements between China and the United States, and found that they were two diametrically opposed policy systems, of which the former followed a top-down process and was in a period of carbon emission intensity reduction, while the latter followed a bottom-up process and was in a period of reduction in total carbon emissions [14]. Some scholars have focused on the carbon trading approach, which could reduce the carbon footprint by purchasing carbon offsets, and has the characteristics of low cost and high efficiency; thus, the improvement of the carbon trading market is an effective way to achieve “carbon neutrality” [15]. Other experts see a path to achieve carbon peaking and carbon neutrality by accounting for the carbon footprint of the whole life cycle of products or the carbon emission processes directly and indirectly related to activities [16,17]. In addition, the low-carbon transformation of energy and industry is also a key method to promote carbon neutrality. Verhagen et al. explored the material requirements and environmental regulations needed for the transition of future urban low-carbon heating technologies [18]. Combing through the existing literature, scholars have paid more attention to carbon-neutral approaches, technologies, fuels, and policies, such as carbon taxes and carbon markets [19], carbon capture and storage technologies [20] and negative-carbon technology [19], renewable clean energy [21], hydrogen and fuel cells [22], carbon footprints and low-carbon transformation [22], and emission reduction policies and mechanisms [20]. In addition, some scholars have also studied research progress, hotspots, and future development trends of carbon neutralization, but they were limited by the small data timespan and the small number of studies [23,24]. Other experts have also taken representative universities and higher education institutions as research cases to analyze the unique initiatives and perspectives of research institutions on carbon neutrality [25]. Wei et al. conducted spatial and temporal research on the development direction of key industries in the field of carbon peaking and carbon neutrality, and identified specific measures for carbon reduction [26]. However, in general, there are still few related studies on the development process, geographical distribution, citation literature, and subject categories of this field, so it is necessary to conduct an in-depth and detailed bibliometric analysis of the development of research in this field. Compared with traditional types of review, bibliometric reviews can identify new growth points in specific fields by studying their developmental characteristics, cross-influence, and penetration relationships.

Based on 4922 studies from the Web of Science Core Collection (WOSCC), this paper used bibliometric software to extract key information from titles, keywords, and abstracts using the LLR, LSI, and MI algorithms to analyze the international research trends on carbon neutrality [27,28]. The comprehensive interpretation of the research hotspots, development context, and future trends in the field of global carbon neutrality from 1991 to 2022 will help scholars and governments to gain insight into the challenges that China will face in the process of achieving carbon neutrality. Additionally, the action will also help experts to elaborate deeper solutions at the macro, meso, and micro levels, having the effect of facilitating the translation of government commitments into corporate actions and conscious public behaviors.

## 2. Materials and Methods

### 2.1. Materials

The WOSCC is considered to be a scientific statistical database that is able to effectively guarantee the authority of data selection [29]. The WOS has become the “trump card index” recognized by scholars in most countries because of its wide range of disciplines and journals [30]. The search formula was as follows: topic search (TS) = (carbon neutrality) or (carbon neutralization); the document types were articles and review articles; and the language was English. Before 1991, the number of publications was relatively small and the annual publication volume did not exceed 3, so this period was excluded from the time range in this article. Finally, the selected timespan was 1991–2022 (as of 31 August), and 4930 records were retrieved. To ensure the comprehensiveness of the data, 4922 documents were obtained after removing duplicates.

### 2.2. Methods

**Figure 1:**
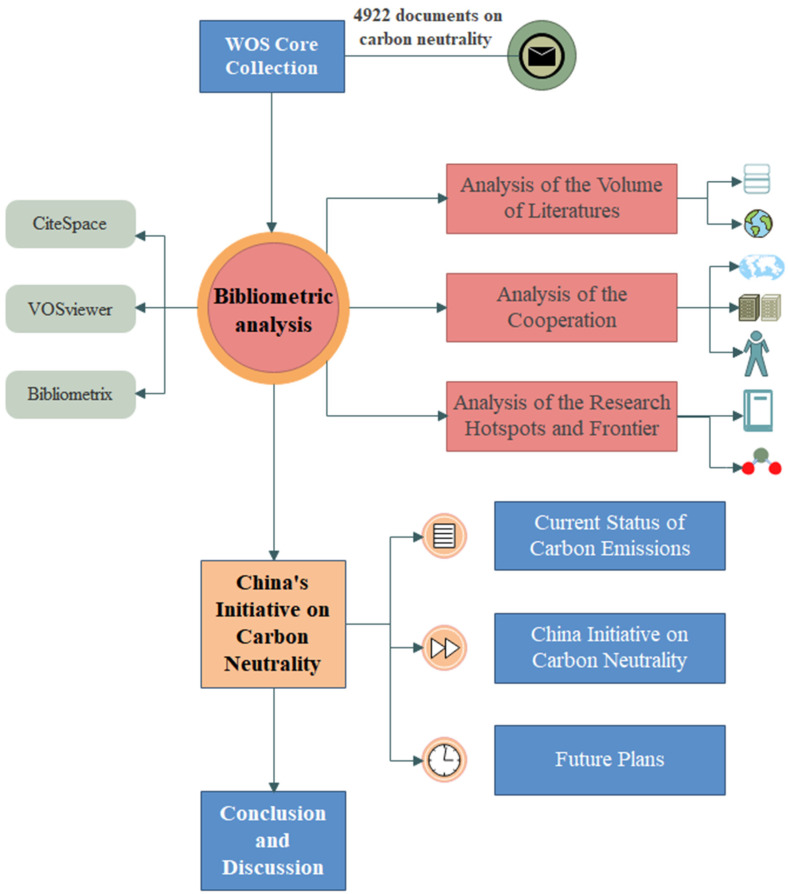
Framework and structure.

This study conducted bibliometric analysis using CiteSpace (6.1.R2 Advanced) and VOSviewer (1.6.18) software and Bibliometrix functions [31]. CiteSpace and VOSviewer are developed based on the WOSCC data format with plain text, enabling analyses of the cooperation network, co-occurrence cluster, and co-citation [32,33]. The method is a combination of mathematics, statistics, and philology, placing particular emphasis on quantifying studies and their content information, and literature-related indicators [34,35]. The bibliometric method is mainly used to study the development characteristics, cross-influence, and penetration relationships of a specific field, so as to determine the new growth point of a specific field [36]. Using visualization methods such as figures and tables is useful for describing the hotspot distribution in the research field of carbon neutrality, and conducting an overall and comprehensive quantitative analysis. The framework and structure of this paper are shown in Figure 1.

## 3. Results

### 3.1. Analysis of the Volume of Literature

#### 3.1.1. Annual Trend of Overall Publishing Volume

**Figure 2:**
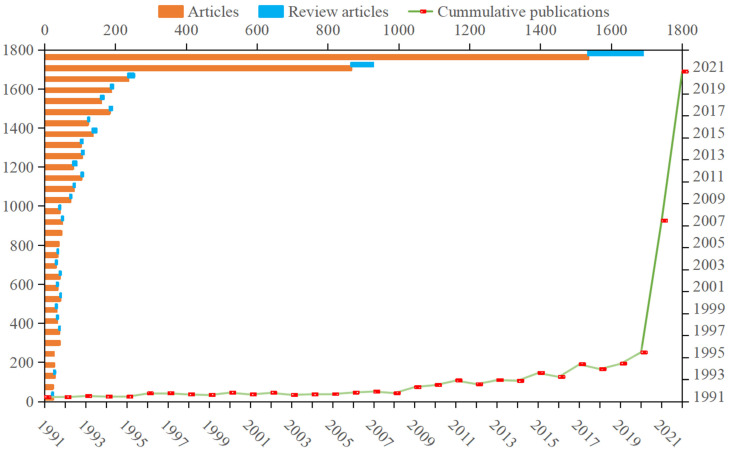
Annual time trends in publications on carbon neutrality.

The annual increase in the number of articles and review articles objectively reflects the attention of scholars and experts, and shows the clear position of the research status and development of a certain field [37]. The statistics of the number of papers published on carbon neutrality by the time distribution are plotted in Figure 2. A total of 4609 articles (93.64%) and 313 review articles (6.36%) were included in the 4922 documents retrieved.

The figure above shows that, while there were more than 150 papers per year on average, the number of global publications on carbon neutrality has been relatively small over the past two decades. The carbon neutrality research can be divided into three stages: In the first stage (1991–2010), it was in its infancy, where the annual publication volume went up from 24 to 85, but the quantity was still not more than double digits. Although it was subject to the United Nations Framework Convention on Climate Change (UNFCCC) in 1992, in general, scholars at this stage paid less attention to carbon neutrality and did not conduct much research [38]. The second stage (2011–2020) was the development period, where the number of documents maintained steady growth, with a total of 1488 articles, accounting for 30.23%. In 2011, the proposed policy of gradually establishing a comprehensive and multi-level pilot of a carbon emission trading market had attracted wider attention among experts and policy decision makers [39]. The third stage (2021–2022) was the explosion period, with 866 and 1534 articles published in 2021 and 2022, respectively, accounting for 48.68% of the total. As with the primary studies, review articles have risen rapidly. Recent reviews (2021–2022) represented 69% of the total number of reviews and 4.39% of the total literature. President Xi Jinping proposed the “30–60” carbon peaking and carbon neutrality goals on 15 March 2021, which heated up the action [40]. Scholars and experts in various industries began targeting carbon neutrality, with some labeling 2021 as the “first year” of the carbon neutrality campaign. The Leaders Summit on Climate in April 2021 prompted all countries to increase their commitment to reduce emissions, and global carbon neutrality has since gradually become an international race. As a top-level design priority, carbon neutrality was added to the process of economic development and social progress, and the construction of clear waters and green mountains [41]. It is necessary to clarify the specific plan and route of carbon neutrality work. High-carbon industries, such as energy demand-side industries, e.g., industry, transportation, and construction, and energy supply-side industries, e.g., electricity, exert joint efforts and take low-carbon action by combining technology, finance, and other means [42,43].

#### 3.1.2. Annual Trend of Published Documents in Top Three Countries

**Figure 3:**
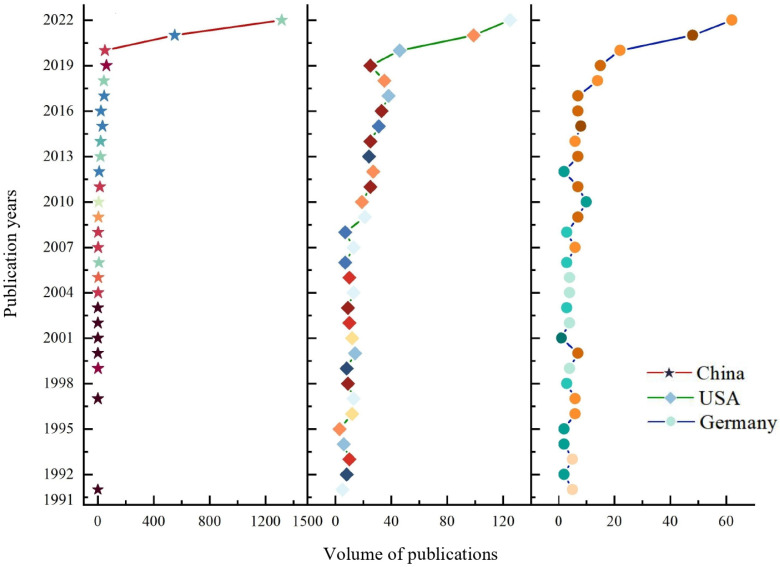
Temporal trends in publications in the top 3 countries (different colors represent different years).

Looking at the annual change in the volume of published documents from a country perspective, the annual trends of the top three countries were consistent with the trend of the total volume of publications in this field (Figure 3). China, the USA, and Germany were at the forefront in terms of the number of published studies, of which 2232 were published by China, 742 by the USA, and 292 by Germany, accounting for 45.27%, 15.05%, and 5.92% of the total number, respectively. The sum of the literature from China and the USA amounted to more than 60% of the total; hence, they were the main exporting countries of carbon neutrality research. 

The volume trend of the literature in China changed frequently, keeping up with the overall international trend. In 2021, Chinese scholars published 550 articles, with an average of 46 articles per month, equivalent to the annual output in 2020. As of August 2022, the number of published articles exceeded 1300, and the average monthly publication volume was 165, which was 3.6 times that of 2021. In 2022, the convocations of the Energy Low-carbon Development Forum (Taiyuan), the 2nd China Carbon Neutral Summit (Shanghai), and the China Carbon Peaking and Carbon Neutrality Summit Forum 2022 (Beijing), as well as the calculation of China’s carbon-neutral development power index, promoted exchanges and cooperation among research teams.

The history of carbon neutrality in the USA could be traced back to the introduction of the Clean Air Act in 1963, so its research efforts were far higher than China’s in the early days, but China far exceeded the number of documents issued by the USA in 2021 [44]. Although the amount had increased in the past two years, the growth intensity was modest. The proposed “3550” plan allowed scholars to conduct research in key areas such as infrastructure, clean energy, electrification, energy conservation and efficiency improvement, and CO_2_ removal [45]. In 2022, there were 125 articles, which did not exceed the average monthly number in China.

Compared with China and the USA, Germany’s annual publication volume was relatively small, accounting for only 5.9% of the total. This is due to the fact that Germany was affected by Russia’s “gas cutoff” and did not have enough natural gas for heating, and the government had to restart coal-fired power stations, which would produce carbon emissions [46]. The policy was contrary to the previous goal of carbon neutrality, but it was a necessary move. Accordingly, Germany delayed its carbon neutrality target by 10 years and is now projected to reach the target in 2045 [16].

### 3.2. Analysis of Cooperation

Complex scientific research, rigorous academic research, and comprehensive knowledge systems have forced experts and scholars to increasingly cooperate and communicate, abandoning the previous independent or small-scale research. In addition, cross-disciplinary research on a certain topic or objective has provided a path for seeking new research growth points, which has gradually become a respected method for scientific research collaboration in academia [47]. The collaboration generally entails greater intensity and broader directions of cooperation, addressed to the macro, meso, and micro dimensions [48]. The macro dimension is the cooperation between countries and regions. The meso level is the internal collaboration of institutions and the support of various types of funds. Additionally, the micro angle is the internal and international collaboration between individual or multiple authors. This paper intended to show the collaboration on global carbon emission policy and research from the macro, meso, and micro perspectives.

#### 3.2.1. Macro-Dimension Cooperation Analysis

**Figure 4:**
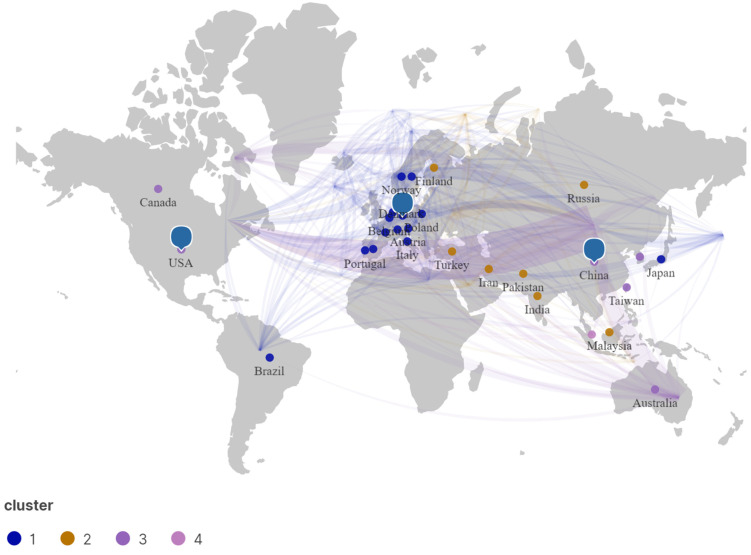
Cooperation between major countries and regions (the blue symbols are the positions of top 3 countries, which are China, USA and Germany).

The intensity of cooperation between countries/regions in terms of carbon neutrality from 1991 to 2022 was visualized using Vosviewer and Scimago Graphica (Figure 4). The size shows the weight of the country’s documents, and the width of the connection indicates the intensity of cooperation between the two countries [49]. To make the map clear, the minimum number of documents of a country was set to 37, ending up with 30 countries. As can be seen from the figure, China and the USA are part of the same cluster, and their nodes are the largest, showing that they were the core countries of carbon neutrality research. There was more cooperation between China and the USA, so the connection is also the strongest. Among them, China (including Taiwan) had the highest number of articles, with a citation frequency of 20,805 and a total link strength of 771. The frequency of citations in the United States was 14,958 higher than that in China, reflecting that the scientific research strength and scientific research level of carbon neutrality were at a high level and had been widely recognized by national scholars. Germa I have checked and revisedny belongs to cluster 1 and forms a large cluster together with European countries. Overall, the results can be divided into two types: developing countries such as Asian countries represented by China and Russia, and developed countries led by the USA and EU, which demonstrate great differences in the quality and frontiers of scientific research.

**Figure 5:**
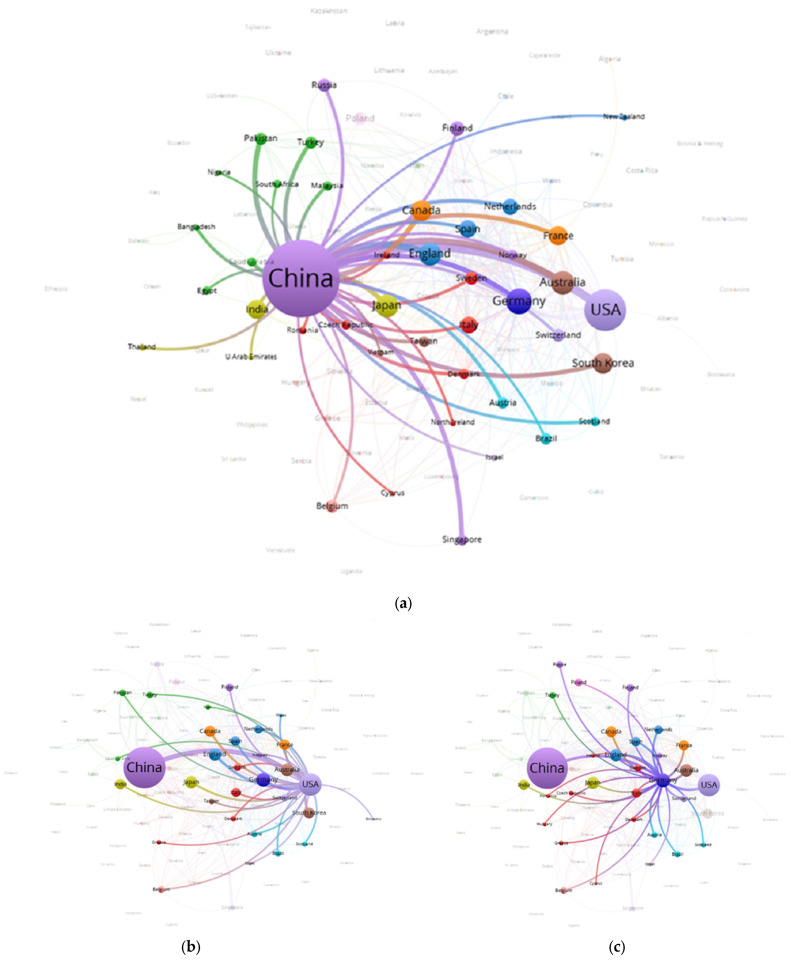
National cooperation networks ((**a**) China; (**b**) USA; (**c**) Germany).

Several detailed cooperation networks between China, the USA, and Germany and other countries are displayed in Figure 5. More than 50% of the cooperation networks of the three countries overlap, indicating that the degree of cooperation was relatively high. Yet, despite that, the cooperation was mainly distributed in several countries, and the scope of cooperation needed to be improved. There were relatively many countries that cooperated with China, so that they were capable of better playing their institutional advantages to promote domestic and national carbon emission processes, and to find a feasible path for developing countries to achieve carbon neutrality. In general, the USA and Germany possessed a smaller difference between the cooperation networks, which is due to the fact that they are both developed countries and have similar stages of economic development. They’re existed other similar situations; a case in point is coal power, which accounted for about a quarter of the energy structure (Germany, 27.1%; USA, 20%), so policy-makers and researchers seeking pathways to achieve carbon neutrality could learn from each other.

#### 3.2.2. Meso-Level Cooperation Analysis

**Figure 6:**
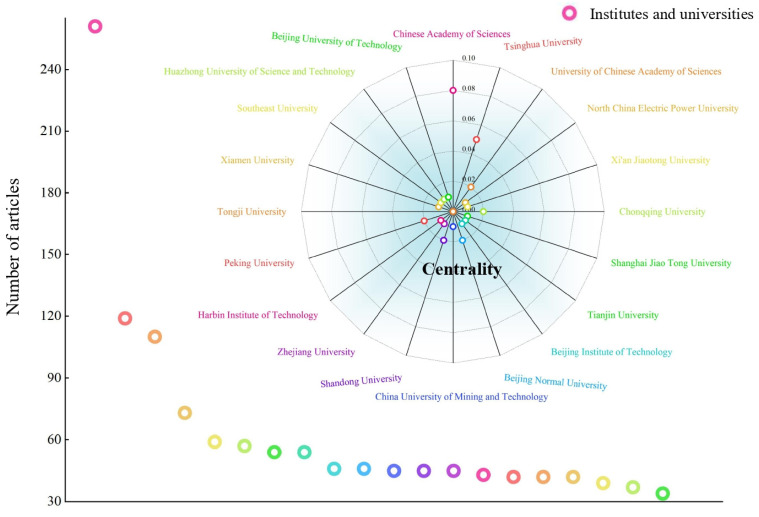
Top 20 institutes and universities.

The map with the number of publications and the degree of centrality was plotted through the Bibliometrix function to analyze the resulting institutional data (Figure 6). There were 19 universities and only the Chinese Academy of Sciences research institutes among the top 20 institutions, which were all Chinese universities or research institutes. They were equally divided into the central and eastern regions of China, especially in cities with rapid economic development, such as Beijing. Among them, Chinese Academy of Sciences ranked first in terms of the number of articles published (261 articles) and the degree of centrality (0.08). Its research on carbon neutrality has been carried out since 1999, with a relatively mature knowledge system and a complete scientific research team. Other universities started their research much later. For example, North China Electric Power University, Beijing Institute of Technology, China University of Mining and Technology, and Beijing University of Technology have only published carbon neutrality articles since 2021. They need to strengthen their cooperation with Chinese Academy of Sciences and internationally renowned research institutes and universities. Through the comparative study of domestic and foreign institutions, it was found that, in terms of the number of documents, most of the high-issuing institutions are well-known research institutes and universities in China, proving that carbon neutrality has aroused widespread concern among Chinese scholars, but there has been a lack of cooperation and exchange with foreign institutions.

The centralities of the Chinese Acad Sci and Tsinghua Univ ranked in the top two, with 0.08 and 0.05, respectively, which explained that they, as the main research organizations in the field of carbon neutrality, had a strong research output capacity. Other universities’ centralities were distributed between 0 and 0.02, and their low index illustrated that these institutions were not the hub organizations in the carbon neutrality field. In the future, the research on carbon neutrality needs to be improved, and focus should be placed on interdisciplinary exchanges and the exploration of new perspectives, new methods, and new policy guidelines. Inter-institutional cooperation mainly occurred between universities, and between universities and research institutes, and there was a lack of exchanges between universities, enterprises, and government departments [50]. The next step is to strengthen the coordinated development of universities, scientific research institutes, enterprises, and government departments, encourage the joint training of highly educated talents, and accelerate the transformation of scientific research results. At the same time, it is necessary to strengthen cooperation and discussion with other well-known international research institutes and universities such as the University of Oxford and the University of Sao Paulo, to work together to address global climate challenges, and to create an atmosphere of international cooperation.

**Figure 7:**
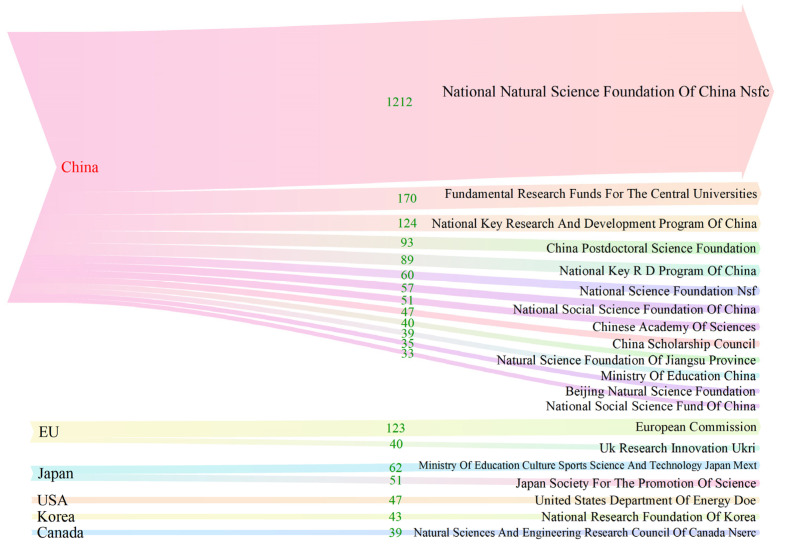
Major funding agencies.

As an important source of funding for major scientific research projects, the funding agencies provided financial assistance for the development of projects and the publication of scientific research results, linking basic research results with social impacts [51]. In addition, they were also conducive to curbing academic misconduct and improving the level of innovation, meaning that Chinese scholars could better gain international academic recognition in terms of research credibility. From this perspective, the obtained data were analyzed to construct a map of major funding agencies (Figure 7).

The above figure shows the top 20 supporting funds, with 2455 articles, accounting for 49.87% of the total of 4922. From the perspective of regional support, China’s distribution of funds was the highest, accounting for 50.37%. The National Natural Science Foundation of China (NSFC), as a national institution, was associated with 31.28% of the publications, closely following the pace of national development and playing a guiding role as one of the important funds for basic scientific research. However, there were only two provincial funding agencies, for whom the number of publications was small. The proportion of the Natural Science Foundation of Jiangsu Province was 1.03%, and the Beijing Natural Science Foundation’s ratio was 0.9%. The rest of the countries were developed regions or countries, namely, the EU, Japan, the USA, Korea, and Canada. It is necessary to open up more channels for cooperation between provincial-level funds, build a national and local integration funding system, strengthen joint cooperation with foreign funds, improve the overall layout of international cooperation, and carry out cooperative research projects, exchange and talent projects (laying the foundation for substantive cooperation), and foreign youth projects (for foreign young scholars), so as to provide strategic support for the output of research results and international cooperation funding.

#### 3.2.3. Micro-Angle Cooperation Analysis

**Figure 8:**
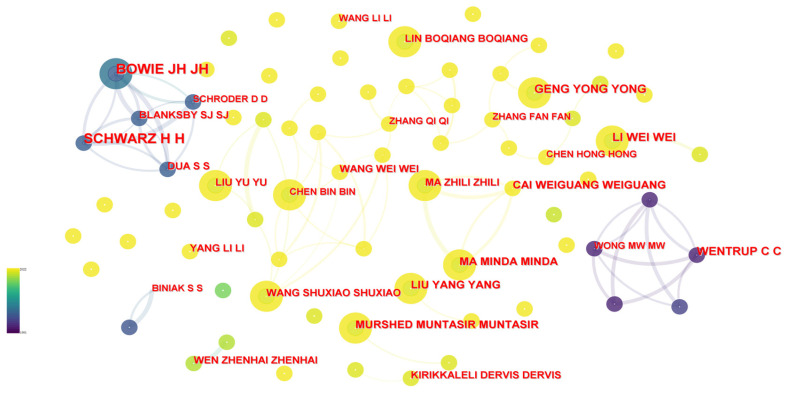
Knowledge map of author cooperation.

The network node was selected as “Authors” in CiteSpace, and then the authors and their cooperative visualizations were initially analyzed, but the resulting map was relatively dense, and there were problems with a lack of focus. In order to make the network more readable, this paper again selected the Minimum Spanning Tree (MST) to prune the network [52]. The algorithm could more vividly represent the field of carbon neutrality research without changing the node. As shown in Figure 8, international author collaboration in the field of carbon neutrality from 1991 to 2022 is indicated from deep blue to light yellow. A total of 1047 authors were obtained, and the density was 0.0012, showing that this value was relatively small, which made it clear that a dense core group of authors had not yet formed among authors. The core authors of a certain field were judged in accordance with Price’s law, and the formula was as follows:

$$m\approx 0.749\times \sqrt[{2}]{n_{max}}$$

where *m* is the minimum number of posts for core authors, and $n_{max}$ is the maximum number of posts among authors. From the statistical results, it can be seen that the maximum number of Schwarz H. articles was 16. Therefore, the calculation was obtained as *m* ≈ 2.996, that is, the number of articles published has to be more than or equal to 3 to be identified as a core author in this field. A total of 178 core authors in this field were identified, with publications contributing 14.89% of the total literature. The authors shown in Figure 8 were all core authors in the field.

The authors were selected based on the top 10 authors in terms of publication volume through burst analysis, and Table 1 was obtained. Through the research, the authors could be divided into two categories: One was the authors who initially attracted attention to the concept of carbon neutrality, but had not published recently, including Schwarz H., Bowie J.H., Wentrup C., and Blanksby S.J. They concentrated on climate change, carbon dioxide emissions, and energy and the linkages between them. The energy industry was also the largest sector in terms of carbon dioxide emissions, where the excessive and prolonged emission of carbon dioxide has caused long term climate change; the pressure to minimize global warming and future extreme weather events has led to higher requirements for renewable and sustainable energy. The other category was the authors who had only been studying carbon neutrality for nearly two years and posted more than four articles annually, and their strengths were 0. The representatives were Li Wei, Geng Yong, Murshed Muntasir, Ma Minda, Liu Yang, and Cai Weiguang. They mainly paid attention to China’s carbon neutrality strategy and action, institutional logic, regional capacity assessment, low-carbon material utilization, and renewable energy technology innovation, and scientifically planned a carbon neutrality path for China to achieve characteristic low-carbon transformation and sustainable development. Although there was cooperation between them, the frequency of cooperation was too small, so cooperation between authors should be strengthened in the future.

Relying on several results, it was found that China should expand the scope and deepen the depth of its cooperation at the level of national cooperation. The larger numbers of documents published mainly came from universities and scientific research institutions, so encouraging enterprises and governments to participate in academic frontier research is quite important. However, the integration of science and education has promoted innovation and development. The maximum number and quality of articles published by individual authors need to be improved, and the formation of a core author group should be accelerated. At the same time, attaching importance to exchanges with internationally renowned universities, understanding their research trends, and learning from available experience are all necessary directions.

### 3.3. Analysis of the Research Hotspots and Frontiers

#### 3.3.1. Analysis of the Research Frontier in Keywords

**Figure 9:**
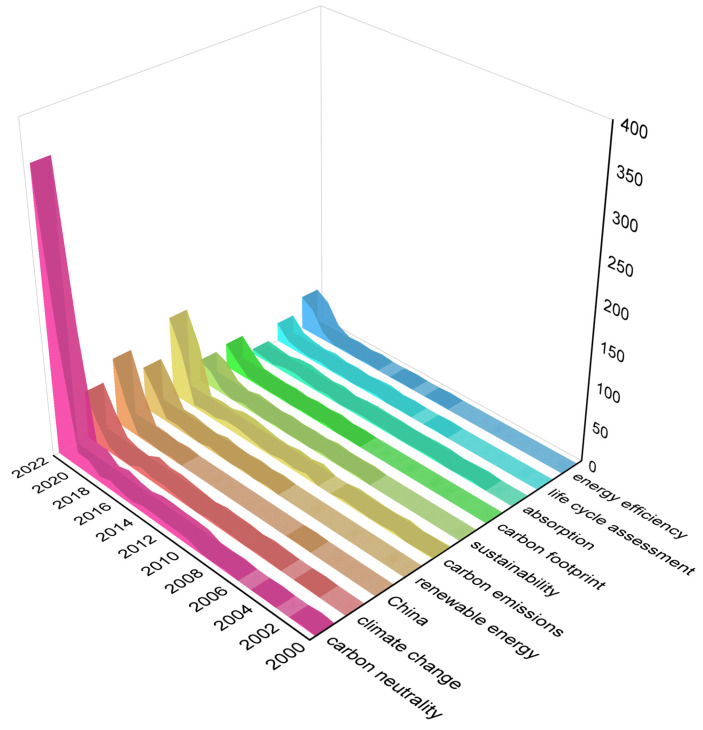
Frequency of the main keywords.

In metrological analysis, the development trends and research frontiers in some fields are usually studied by extracting the number of occurrences that the keywords or subject words appear in the analyzed data. In this paper, the Bibliometrix function in the R language was used to obtain the top 30 keywords, and then the synonyms function was used to merge repeated words. Specifically, carbon neutrality = carbon neutral = neutralization = carbon neutralization, carbon emissions = carbon dioxide = CO_2_ emissions, sustainability = sustainable development, energy efficiency = energy transition. The merged data were filtered, and the years where the occurrence frequency was almost 0 before 2002 were deleted; then, the keyword frequency map from 2002 to 2022 was obtained. Figure 9 reveals the top 10 keywords in terms of frequency, most of which were related to the topic of carbon neutrality before 2010 that had not attracted the attention of scholars. Relatively speaking, there were many papers related to carbon emissions during this period, focusing on the synthesis of carbon materials in the field of chemistry. Regarding “carbon neutrality”, the frequency did not exceed 2 before 2009, and the average number of occurrences from 2010 to 2018 did not exceed 9. A small increase began in 2019, rising to 153 in 2021, and reaching up to 348 in 2022. Ranked by keyword intensity were carbon neutrality, carbon emissions, China, climate change, renewable energy, energy efficiency, carbon footprint, sustainability, life cycle assessment, and absorption. Scholars often choose the concepts of “life cycle assessment” (LCA) and “carbon footprint” (CF) when accounting, reducing emissions, and disclosing carbon neutrality. Carbon emissions accounting was the basis for all carbon neutrality efforts, and the scope included direct emissions, indirect emissions from energy production, and indirect emissions from raw material production divided by life cycle. The LCA method contained a variety of resource and environmental indicators, and later scholars had taken into account climate change factors to add the carbon footprint of all products of a company together to develop an ISO standard to form CF. Ming Bao et al. quantified the CF of green tea through LCA, and conducted a study of carbon neutrality pathways in the whole chain of tea production in 16 major tea-producing regions in China [17]. Jichao et al. used the net carbon sequestration rate of paddy fields to analyze the current situation of carbon neutrality in China, and it was found that carbon dioxide emissions could be controlled by means of water conservation and biochar [53].

**Figure 10:**
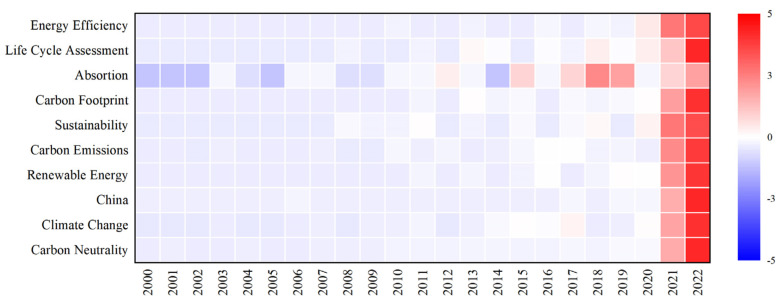
Heat map of keywords.

The data in the graph were normalized using the z-score, and the average value of the frequency of keyword occurrences in the same sample was used as the benchmark to obtain a keyword heat map. In Figure 10, values higher than the mean are positive, marked in red, while values below the mean are negative, marked in blue. We found that the top 10 keywords appeared more frequently in the past two years, especially in 2022, and the frequencies of other years were blue and slightly lower than 0. It could be predicted that research topics on carbon neutrality will continue to be hotly debated in the academic community, and more accurate and detailed solutions will be introduced to develop more effective and energy-saving technologies for low carbon and zero carbon. In the past two years, scholars have conducted research on carbon neutrality from different perspectives, especially renewable energy, carbon emissions, carbon footprint, sustainable development, energy efficiency, etc., but there were relatively few studies on carbon absorption. Renewable energy contributed to the goal of “carbon neutrality” in terms of clean substitution, mainly from the two perspectives of clean power generation and direct utilization, and explored emission reduction technologies for six resources, such as wind, water, nuclear, solar energy, biomass, and geothermal. Achieving carbon neutrality is related to people’s well-being, economic prosperity, and sustainable ecological development, and can fundamentally realize green and low-carbon sustainable development.

#### 3.3.2. Analysis of the Research Frontier in Subject Categories

**Figure 11:**
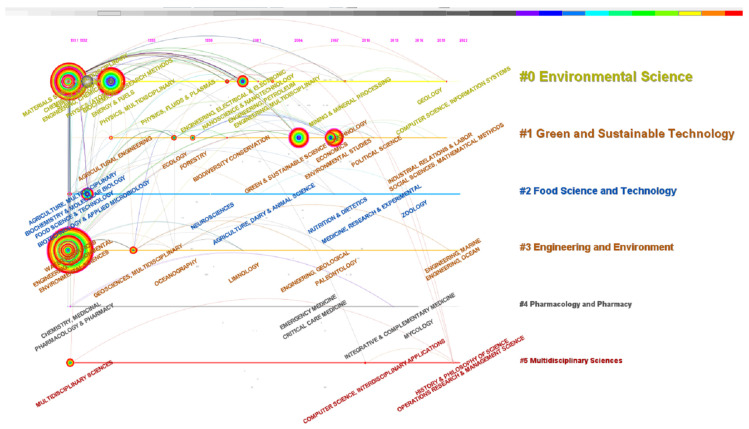
Timeline graph of categories.

This paper extracted information from the SC field (research direction) and then selected “category” as the source to generate a domain timeline map (Figure 11). Since 1991, scholars have been paying attention to the two major categories of #0 environmental science and #3 engineering and environment, and close relationships have existed between the two clusters. Environmental science has exploded from 58 in 2020 to 572 in 2022. Among them, the highest citation frequency for an article was 1157. Kondratenko et al. studied the application of carbon dioxide and hydrogen energy in energy and chemicals production, and effectively converted CO_2_ into value-added chemicals under photocatalytic and electrocatalytic technologies [54]. The occurrence frequency of engineering and environment had increased from 19 in 2020 to 161 in 2022. Many studies concerned the use of polytitanium tetrachloride (PTC), ferric chloride (FeCl3), and titanium tetrachloride (TiCl4) in various types of wastewater treatment to improve coagulation performance and flocculant recovery, where the purpose was to find an effective coagulant to achieve waste recycling [55,56]. In addition, #2 food science and technology was another category that experts have long valued. The agricultural food industry is crucial to human life and has a complete industrial chain of research and development, breeding, processing, packaging, logistics, distribution, retail, and storage. In order to achieve carbon neutrality, it is necessary to implement different carbon neutrality measures at different stages of the industrial chain, such as improving the efficiency of chemical fertilizers, recycling methane in manure, using clean energy, utilizing recyclable materials for packaging, and reducing carbon emissions in transportation and distribution. #1 Green and sustainable technology was also a major theme; after all, carbon neutrality and sustainable development complement each other, being based on the scientific evaluation of the existing environment. Carbon neutrality is an objective need for sustainable development, and sustainable development is the proper goal of carbon neutrality.

#### 3.3.3. Analysis of the Research Frontier in Literature Co-Citation

**Figure 12:**
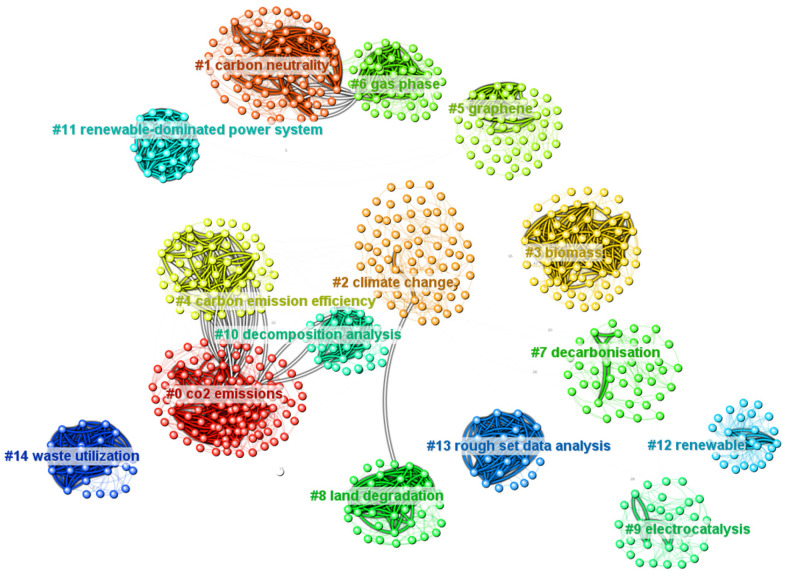
Graph of literature co-citation.

This paper used CiteSpace, known as the “citation space”, to analyze the co-citation, combining the cited literature (TI field) with the reference literature (CR field), to find the landmark literature, and to explore the research frontier and knowledge base of this subject. The term “research frontier”, proposed by Price, can be used to show that the articles in the network with high citation and strong timeliness could dynamically reflect the hot changes in this field by combining the intermediary centrality, degree, and highlight points [57]. CiteSpace was used to extract cluster labels from the keywords of the cited articles to form Figure 12 by establishing connections in the cited literature and references.

Co-citation analysis of the literature based on keywords found that #0 CO_2_ emissions, #1 carbon neutrality, #2 climate change, and #4 carbon emission efficiency were consistent with the hotspots obtained in the above section. The density of the wires indicates the degree of association of the core literature within or between different clusters. As can be seen in the figure, the intra-cluster connection was denser than between clusters, showing that the same topic was more vulnerable to the attention of scholars and could easily form a large number of core studies. The connection between #0 CO_2_ emissions and #4 carbon emission efficiency was the densest, and scholars often considered carbon dioxide in conjunction with efficiency. There were also thicker connections between #1 carbon neutrality and #6 gas phase. Shan et al. published articles with the highest number of citations at 49, with a degree of 11 and centrality of 0.01. Shan et al. used the emission accounting method of IPCC to account for energy and process-related emissions in various provinces in China, in order to provide data support for emission reduction actions [58]. The degree measures the external connectivity of a node. Ji et al. had the highest degree of 28. Ji, X. et al. compared the differences between China and the BRICS countries in terms of carbon-neutral and related investments such as green funds and high-emission funds, and assessed China’s future carbon neutrality and economy [59].

## 4. Discussion

**Figure 13:**
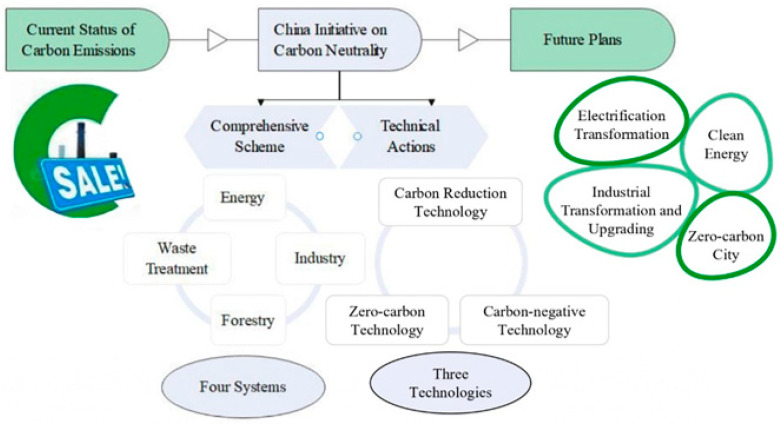
China’s road to carbon neutrality.

Achieving carbon neutrality plays a key role in adapting to the sustainable development of China’s ecological construction, assuming national responsibility for climate change, promoting efficient energy emission reduction, and promoting green and low-carbon economic development. However, the process is challenging and the task is daunting. This paper analyzes China’s road to carbon neutrality from the perspectives of the current status of carbon emissions, carbon neutrality initiatives, and future plans, as shown in Figure 13.

### 4.1. Current Status of Carbon Emissions

The government has actively responded to climate change, issued various schemes, accelerated the pace of energy structure adjustment, established low-carbon demonstration pilots, and achieved results in green and low-carbon development. China’s emission reduction measures are equivalent to reducing carbon dioxide by about 5.6 billion tons, accounting for half of the overall emissions [60]. Eight carbon emission trading market pilots were established to constrain the carbon behavior of enterprises and individuals through policy tools such as carbon taxes, carbon allowances, and carbon credits [61]. At the same time, China actively participated in world energy governance, carried out South-South cooperation and exchanges, and implemented the 2030 Agenda for Sustainable Development.

However, it can be predicted that, although China’s economic growth rate would show a steady downward trend (2020–2035), the development model of being highly dependent on fossil energy would also not change immediately, inevitably producing a large amount of carbon dioxide. Energy was a major part of emissions, accounting for 80%, but clean energy accounted for only 15% of it [62]. The World Energy Outlook 2019 mentions that the proportion of carbon-neutral clean energy needs to reach more than 65% [63]. The heavy reliance on coal in some sectors has created difficulties for emission reduction work. China has developed from the late stage of industrialization to the post-industrialization stage, and the carbon dioxide emissions of industry and electricity are at the forefront, accounting for 85%. In recent years, agriculture, construction, and chemical industries have gradually become carbon-neutral. Low-carbon, zero-carbon, and negative-carbon technology has been at a low level, the cost has been high, and the potential for emission reduction and application was faced problems of interoperability.

### 4.2. China’s Initiative on Carbon Neutrality

#### 4.2.1. Comprehensive Scheme

Carbon neutrality is mainly achieved through action in four areas: energy system, industrial system, forestry, and waste treatment [64,65,66]. Among them, the energy system and industrial system are the leaders in carbon emissions, forestry has a negative effect, and the proportion of carbon neutral waste disposal is extremely low. The energy system can mainly reduce emissions through carbon removal, and advocating the combination of clean and electric energy substitution. Industrial systems rely on increasing energy efficiency, focusing on the reduced use of raw materials and fuels, and developing low-carbon products. Forestry increases carbon sinks by expanding the acreage area.

It can be roughly divided into three stages: The first stage is the peaking stage (before 2030), where the peak is controlled at about 11.5 billion tons. The core measures are to control and reduce coal emissions, attach importance to clean energy to make up for the lack of supply, achieve peaks in energy production and use, and increase the contribution of independent emissions. The second period is the carbon reduction stage (2030–2050). The core measures are to improve the energy allocation network, reduce the power system to nearly zero emissions, reduce emissions to 1.38 billion tons, vigorously develop clean energy, and improve zero-carbon and negative-carbon technology. The third stage is the neutralization phase (2050–2060), focusing on forestry and waste disposal, in-depth development of carbon capture and storage, and efforts to achieve negative carbon emissions.

#### 4.2.2. Technical Actions

Firstly, technical actions include carbon reduction technology, involving the coal, electricity, steel, transportation, and other high-energy-consuming industries; methods include process substitution, optimization, energy efficiency improvement, resource recovery, and the development of raw material dye replacement technology, which are key measures to achieve the peak as soon as possible [67].

Secondly, the technology includes zero-carbon technology, mainly referring to clean power generation in various energy storage systems, where clean energy has become the dominant power source [68]. Photovoltaic power generation has focused on improving cell conversion efficiency, and experts have researched new multi-PN junction cascade cells. The photovoltaic modalities in the western region of China have good potential and low development cost, and could be developed on a large scale. Wind power generation focuses on improving the capacity and efficiency of single units, and developing offshore and polar wind power. The average annual wind power density in the western and northern regions was 200 W/m^2^ and rich in resources. Under the condition of ensuring safety, nuclear power improves efficiency, and nuclear fusion technology is being fully grasped. Biomass power generation combines carbon capture and storage to reduce costs and narrow regional gaps.

Thirdly, the science has mainly included negative-carbon technology, including increasing ecological carbon sinks and carbon capture/utilization/storage [69]. Ecological carbon sinks could be increased—from soil improvement to marine utilization. Carbon capture, utilization, and storage (CCUS [70]) includes direct air capture and storage (DACS [71]), bio-energy with carbon capture and storage (BECCS [72]), and carbon dioxide resource utilization. At present, there are fewer BECCS projects, which are mainly concentrated in European and American countries. The cost difference of CCS between different technologies is large, and there are many technical paths to explore, which have great potential for emissions reduction and carbon neutrality.

### 4.3. Future Plans

Carbon emissions are a comprehensive reflection of human economic activities. By reducing the carbon emission intensity and energy intensity, improving energy efficiency networking, and developing carbon removal and negative emissions technology, from the perspective of the energy total, structure, and intensity, we can achieve clean energy production and electrification of energy consumption. We can also seek different carbon-neutral paths across countries, industries, energy systems, and power systems.

The transformation of terminal energy consumption to electrification should be promoted, and high-energy manufacturing industries such as steel, building materials, chemicals, nonferrous metals, and paper should be transformed to low-emission and clean industries. Electric energy is a zero-emission energy source, so we should adhere to replacing traditional fossil energy with electricity, improve the level of electrification, and build a nationwide stable and sustainable power transmission platform. In 2060, the electrification rate will reach 66%, traditional fossil energy will be reduced by about 5%, and hydrogen energy (5%), biomass energy (5%), and other renewable energy (5%) will be developed.

Clean energy should be developed on a large scale; the proportion of solar and wind energy (30% each) should be expanded; allocation efficiency should be improved; stability and storage should be increased; fossil energy dominance (reduce to less than 5%) should be abandoned; and a clean-based energy system should be established.

Industrial transformation and upgrading need to be realized. Emerging and green industries need to lead the development direction, including the low-carbon transformation of high-energy-consuming industries. High-quality development of productive and living service industries needs to be achieved. The low-carbon industry needs to be improved. Transportation needs to be electrified. Zero-carbon building energy efficiency needs to be improved. A comprehensive ecosystem needs to be built.

With points and lines, a zero-carbon city model was comprehensively built to promote it from the three directions of people, production, and domain. The public are the main body of low-carbon action, advocating green travel and commuting, sustainable material application, community garbage classification, and waste recycling. The urban green economy is closely related to the layout of zero-carbon industries, and it is biased towards the intensive development of mixed land use and the comprehensive development of above-ground and underground space to make full use of land resources to build a zero-carbon economic and social system. Green and open public spaces such as low-carbon community parks and large-scale eco-city squares should be constructed, sustainable infrastructure should be improved, and urban ecological efficiency should be enhanced. We need to establish a road network circulation and ecological slow-travel system to control traffic energy consumption. Using digital innovative energy-saving technology, we also need to design urban green energy production and consumption cycle systems, and compress spatial energy consumption.

## 5. Conclusions

In recent years, the rate of China’s surface temperature rise has been about twice that of the world, and it is facing more severe challenges. The proposal for carbon neutrality is a necessary response to climate change. In this paper, we visualize the hotspots, frontiers, and emerging trends of carbon neutrality research in the form of quantitative literature analyses, and sort out their development context from the three sectors of article volume, cooperation, and keywords. This study provides a reference for scholars and decision makers to explore the feasible path of carbon neutrality and formulate relevant policies, as well as helping enterprises accurately grasp the research and development and investment direction of carbon neutrality technology in the future. This article, after a detailed study, draws the following conclusions:

(1) Carbon neutrality research was generally concentrated in the past two years, focusing on the total amount, industry, evaluation methods, technical research, and other aspects. China, the USA, and Germany were the main producing countries of the articles, reflecting the high importance attached to carbon neutrality in both developing and developed countries, and research in this field has broad prospects.
(2) Inter-country cooperation was spread across several major countries, and scholars had a high degree of recognition of international citations. Institutional involvement was concentrated among students and teachers in schools, and socialists, entrepreneurs, and politicians were less involved. Most of the research funds were from national projects because, firstly, relevant research needs strong financial support, and, secondly, local governments have been less concerned than the central government. In the early stage, the leading authors formed a core author group, but in recent years, the main small research teams have not yet formed a large research group.
(3) High-frequency keywords focused on carbon neutrality, carbon emissions, energy efficiency, sustainable development, and other related topics. Additionally, in recent years, research in the field has become more and more abundant, from the fields of environment, science and technology, engineering, and pharmacy to multi-disciplinary integration, showing a multi-level, multi-subject, and multi-system trend. Regarding each of the subject keywords, a large number of authors had studied them, forming a relatively close network of contacts.

In view of the problems and challenges in the research field concerning carbon neutrality in this paper, the following suggestions are made:

Firstly, build an energy Internet and accelerate the low-carbon transformation of energy. Stabilize high-voltage devices for energy transmission, promote the coordinated development of energy in various regions, and open the national networking model. Vigorously develop clean and renewable energy, comprehensively accelerate the development of solar energy, develop wind power intensively and efficiently, develop nuclear power safely and effectively, and attach importance to the development of hydrogen energy and biomass energy.

Secondly, accelerate technology research and development, apply low-carbon, zero-carbon, and negative-carbon technologies to different industries, maximize industry efficiency, carry out collaborative research and development, reduce R&D costs, attach importance to the research of green hydrogen-related materials such as electric hydrogen production or hydrogen steelmaking, and cultivate emerging zero-carbon and green industries.

Thirdly, promote the construction of a unified national carbon market, improve the rules of the carbon trading market, advance the investment and financing mechanisms as well as green credit mechanisms, give play to the supporting role of fiscal policies, and invigorate market vitality.

Finally, advocate a green lifestyle, improve green transportation facilities, promote the construction of zero-carbon communities and cities, and promote the zero-carbon transformation of the whole society.

## Figures and Tables

**Table 1:** Top 10 authors of publications in carbon neutrality (red represents that the author is the core author, and blue represents that the author is a non-core author in this period).

Authors	Published Volume	Strength	Begin	End	1991–2022
Schwarz H.	16	6.29	1991	2000	▃▃▃▃▃▃▃▃▃▃ ▂▂▂▂▂▂▂▂▂▂▂▂▂▂▂▂▂▂▂▂▂▂
Bowie J.H.	15	7.75	1998	2006	▂▂▂▂▂▂▂ ▃▃▃▃▃▃▃▃▃ ▂▂▂▂▂▂▂▂▂▂▂▂▂▂▂▂
Wentrup C.	9	5.43	1994	1997	▂▂▂ ▃▃▃▃ ▂▂▂▂▂▂▂▂▂▂▂▂▂▂▂▂▂▂▂▂▂▂▂▂▂
Li Wei	9	0	2021	2022	▂▂▂▂▂▂▂▂▂▂▂▂▂▂▂▂▂▂▂▂▂▂▂▂▂▂▂▂▂▂ ▃▃
Geng Yong	9	0	2021	2022	▂▂▂▂▂▂▂▂▂▂▂▂▂▂▂▂▂▂▂▂▂▂▂▂▂▂▂▂▂▂ ▃▃
Murshed Muntasir	8	0	2021	2022	▂▂▂▂▂▂▂▂▂▂▂▂▂▂▂▂▂▂▂▂▂▂▂▂▂▂▂▂▂▂ ▃▃
Ma Minda	8	0	2021	2022	▂▂▂▂▂▂▂▂▂▂▂▂▂▂▂▂▂▂▂▂▂▂▂▂▂▂▂▂▂▂ ▃▃
Liu Yang	8	0	2021	2022	▂▂▂▂▂▂▂▂▂▂▂▂▂▂▂▂▂▂▂▂▂▂▂▂▂▂▂▂▂▂ ▃▃
Cai Weiguang	8	0	2021	2022	▂▂▂▂▂▂▂▂▂▂▂▂▂▂▂▂▂▂▂▂▂▂▂▂▂▂▂▂▂▂ ▃▃
Blanksby S.J.	7	3.94	1998	2003	▂▂▂▂▂▂▂ ▃▃▃▃▃▃ ▂▂▂▂▂▂▂▂▂▂▂▂▂▂▂▂▂▂▂

## Data Availability

Not applicable.
